# Case Report: A neurolinguistic and neuroimaging study on a Chinese follow-up case with logopenic-variant of primary progressive aphasia

**DOI:** 10.3389/fneur.2022.963970

**Published:** 2022-09-20

**Authors:** Binyao Huang, Xiaolu Wang, Biao Jiang, Linlin Kong, Haifeng Hou, Jiong Zhou

**Affiliations:** ^1^School of Foreign Languages, Zhejiang University of Finance and Economics, Hangzhou, China; ^2^School of Foreign Languages, Zhejiang University City College, Hangzhou, China; ^3^Department of Radiology, Second Affiliated Hospital, School of Medicine, Zhejiang University, Hangzhou, China; ^4^Department of Neurology, Second Affiliated Hospital, School of Medicine, Zhejiang University, Hangzhou, China; ^5^Department of Nuclear Medicine, Second Affiliated Hospital, School of Medicine, Zhejiang University, Hangzhou, China

**Keywords:** Alzheimer's disease, primary progressive aphasia, logopenic-variant, neurolinguistics, neuroimaging

## Abstract

Primary progressive aphasia (PPA), typically resulting from a neurodegenerative disease, is characterized by a progressive loss of specific language functions while other cognitive domains are relatively unaffected. The logopenic variant, characterized by impairments of word retrieval and sentence repetition along with preserved semantic, syntactic, and motor speech abilities, is the most recently described and remains less understood than other variants due to a comparatively small number of case studies and a lack of investigations with a thorough specification. In this article, we report a 2-year follow-up case study of a 74-year-old Chinese female patient with a logopenic variant of primary progressive aphasia, including its neurolinguistic study, magnetic resonance imaging (MRI), and 11C-Pittsburgh compound B-Positron emission tomography imaging analyses, as well as gene sequencing. This case confirms that, in addition to word-finding and sentence repetition difficulties, the logopenic variant may also present with mild auditory comprehension and naming deficits attributed to impaired access to lexical representations. The observation of clinical treatment suggests the efficacy of memantine hydrochloride tablet and rivastigmine transdermal patch in slowing down the cognitive deterioration of this patient. The description and exploration of this case may shed new insights into a better understanding of the Chinese logopenic variant of primary progressive aphasia.

## Introduction

Primary progressive aphasia (PPA) is a general term for a group of neurodegenerative diseases that primarily affect language processing. Three major variants can be classified based on their aphasia profile and atrophy pattern. The diagnostic criteria of PPA initially only comprised non-fluent/agrammatic variant PPA (navPPA) and semantic variant PPA (svPPA), whereas logopenic variant PPA (lvPPA) has been included only recently (1). Hence, the characterization of lvPPA remains incomplete and calls for a comprehensive description of lvPPA features. The in-depth study of lvPPA is furthermore essential because it contributes to expanding our understanding of the pathological process of Alzheimer's disease (AD), given that some studies have demonstrated a high proportion of underlying Alzheimer's pathology (2).

The concept of atypical forms of AD was recently proposed and precisely elaborated (3), including a posterior variant, a frontal variant, a logopenic variant, and a Down's syndrome variant. To our knowledge, few observations have been made regarding the neurolinguistic features of Chinese-speaking patients with lvPPA and the long-term structural changes of the brain accompanying the progressive course. Hence, patients with lvPPA from Chinese-speaking populations might exhibit some characteristics that should be explored in depth.

## Patient consent

This case report and accompanying images were published with the written informed consent of the patient's guardian.

## Description of the case

**Figure 1:**
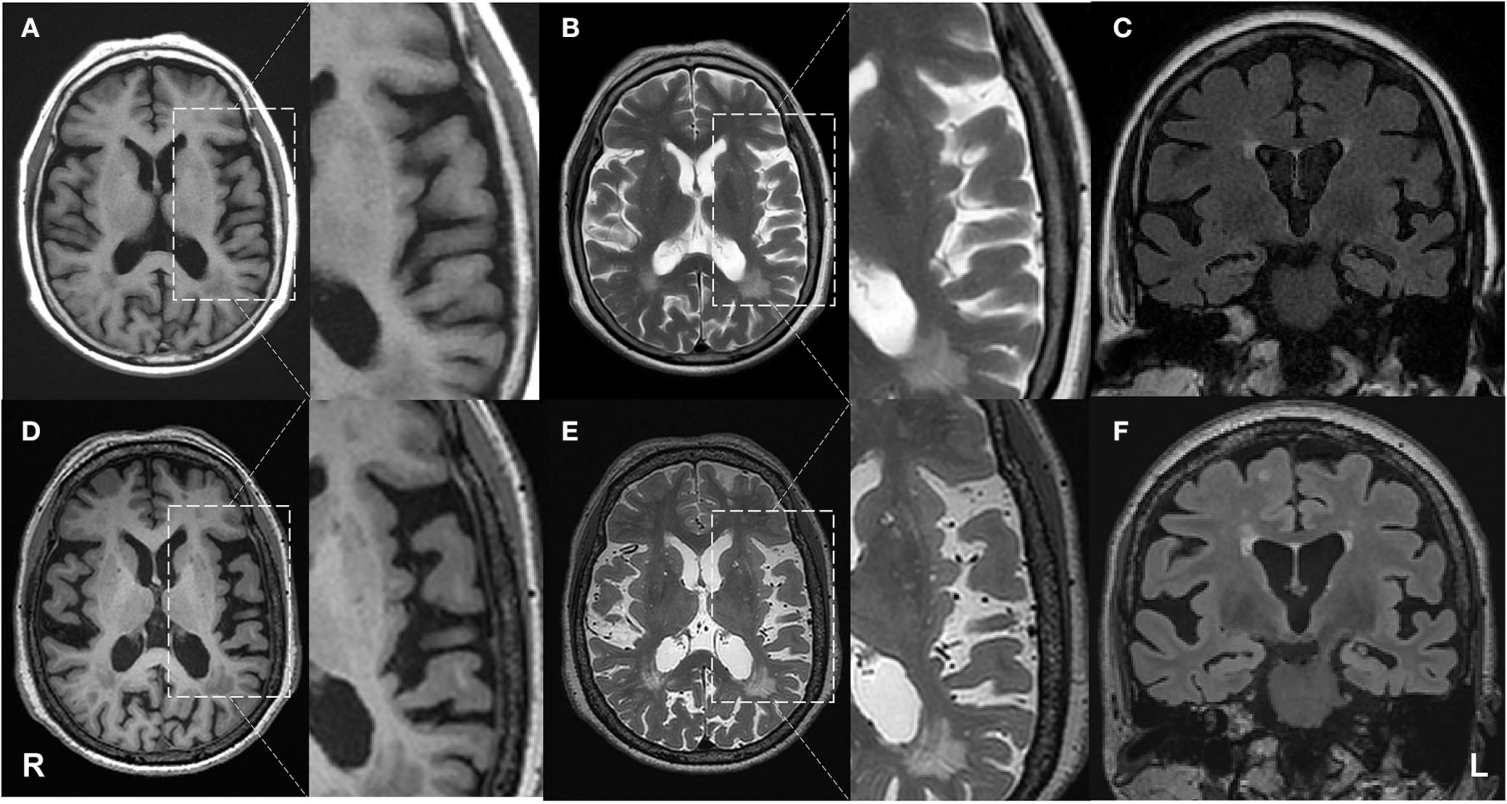
Progressive brain atrophy was detected by structural MRI. First row: MRI on 15 February 2019; second row: MRI on 25 April 2021. **(A,D)** axial T1 weighted images; **(B,E)** axial T2 weighted images; **(C,F)** coronal FLAIR images. The patient showed atrophy in the left temporal lobe compared with the baseline.

Reported here is the case of a Chinese right-handed female retired worker born in August 1948 who has been experiencing progressive language impairment in her native Han language since 2015. The patient has only 2 years of primary education and is virtually illiterate, unable to read or write. On 15 February 2019, the patient visited the memory clinic of our hospital for the first time, with complaints of speech difficulty for 4 years and mild memory loss for 2 years. According to the patient's family, she began having trouble finding words in 2015, which led to stumbling speech with progressive aggravation. In 2017, she started to show signs of memory loss, as evidenced by forgetting to take groceries after paying for them and pressing the button of the corresponding floor again after reaching the target floor by elevator. In 2018, she once went in the wrong direction when going out but never got lost. Her primary symptom in 2019 was word stumbling, which resulted in frequent pauses in her speaking. However, what she said can still be appropriately understood by others. The patient was in good general health except for a 10-year history of hypertension. Her physical examination was normal. Blood tests, such as routine blood examination, biochemistry, coagulation function, thyroid function, and screenings for human immunodeficiency virus (HIV), syphilis, and glycosylated hemoglobin, did not reveal significant abnormalities. The patient underwent brain magnetic resonance imaging (MRI; Simens, 1.5T) on the same day, and two neuroimaging specialists with more than 10 years of experience rated the scans. A mildly widened lateral sulcus was observed, with the left more pronounced than the right (Figure 1). Moreover, the bilateral lateral ventricular horns were slightly broad and blunt, which was evident in the coronal position. Lesions of white matter hyperintensities (WMH) on T2-weighted MRI were seen around the ventricles and under the frontoparietal cortex. The bilateral choroidal fissures were widened. Mild ventriculomegaly was detected on the left temporal horn of the lateral ventricle. The right hippocampal volume (HV) showed I-degree atrophy, while the left HV displayed II-degree atrophy.

**Figure 2:**
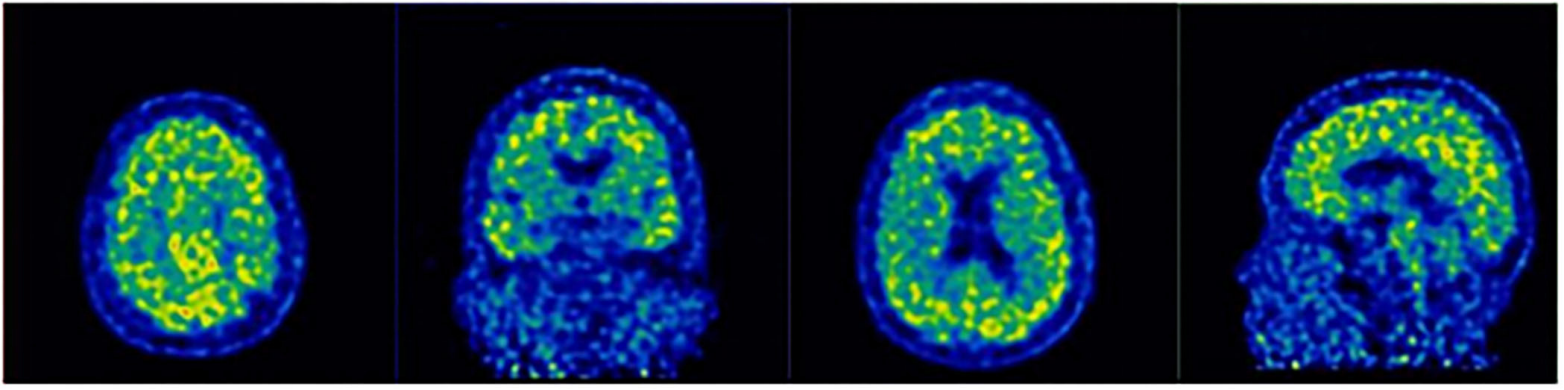
11C-Pittsburgh compound B-Positron emission tomography imaging (PIB-PET) revealed the accumulation of Aβ in the prefrontal cortex, temporal lobe, posterior cingulate cortex, and parietal-occipital junction cortex.

On 21 February 2019, 11C-Pittsburgh compound B-Positron emission tomography imaging (PIB-PET) was performed, which presented a significant increase in amyloid β-protein (Aβ) in the cerebral cortex (Figure 2), including bilateral frontal (SUV_mean_ = 2.50), parietal (SUV_mean_ = 2.86), temporal (SUV_mean_ = 2.30), occipital (SUV_mean_ = 2.25) lobes, and cerebellar cortical (SUV_mean_ = 1.07).

The accumulation of amyloid is related to the evidence of AD, such as the deterioration in cognitive functioning, memory, fine motor movements, executive functioning, and visuospatial skills. Therefore, the patient was diagnosed with AD and began to receive treatment with memantine hydrochloride tablet and rivastigmine transdermal patch. The patient gradually reached the maximum daily dose (20 mg qd) of memantine hydrochloride tablet. The initial dose of rivastigmine transdermal patch was 4.5 mg qd, which was changed to 9.5 mg qd after 1 month.

On 5 August 2019, neurolinguistic and neuropsychological assessments were conducted and reported, including the Mini-Mental State Examination (MMSE) (4), the Clinical Dementia Rating (CDR) (5), the Neuropsychiatric Inventory (NPI) (6), and the Activities of Daily Life (ADL, the 20-item edition) (7) (Table 1). In addition, the patient was evaluated using the Aphasia Battery of Chinese modified by the First Affiliated Hospital of Peking University (ABC) (8). The results are shown in Table 2. The patient's MMSE score was 15 in 2019. She scored on the NPI of 0, CDR of 0.5, and ADL of 23. Moreover, the ABC test for language evaluation showed that her repetition and naming were deficient. Her poor performance in structure and visual space perception may be caused by her low level of education. The assessments also revealed her short-term memory impairment.

**Table 1:** Results of ADL evaluating at age 71 and 73.

**Items**	**First evaluation score**	**Second evaluation score**
Feeding	1	1
Dressing	1	1
Washing up	1	1
Transferring	1	1
Moving around the house	1	1
Toileting	1	1
Help with incontinence	1	1
Bathing	1	1
Using public transportation	2	2
Getting around outside	2	2
Cooking one's own meals	1	2
Taking medications	1	1
Light housework	1	1
Heavy housework	2	2
Doing laundry	1	1
Toenail clipping	1	1
Shopping	2	3
Using the telephone	2	3
Handling finances	2	3
Being alone at home	1	2
Total score	26	31

1: can do it by oneself without any difficulty.

2: can do it by oneself with some difficulties.

3: can do it with help.

4: cannot do it even with help.

**Table 2:** Results of ABC (Aphasia Battery of Chinese) evaluating at age 71 and 73.

**Tasks**	**Items**	**First evaluation score**	**Second evaluation score**
Oral expression	Amount of information	85 PR	80 PR
	Fluency	18/27	18/27
	Serial language	21/21	21/21
	Repetition	57/100	27/100
	Word naming	28/40	26/40
	Color naming	12/12	12/12
	Responsive naming	6/10	6/10
Auditory comprehension	True/False	44/60	44/60
	Auditory identification	73/90	72/90
	Oral instruction	31/80	39/80
Operation		18/30	17/30
Calculation		20/24	8/24
Structure and visual space perception		1/10	1/10

PR, percentage.

We analyzed the recordings of the patient's description of a cookie-theft chart during the first assessment in 2019. Among all errors, the most frequent occurrences are phonetic errors, but semantic errors also occur occasionally. For example, this patient was able to correctly name “*deng zi*” in Chinese (“stool” in English), which proves her intact object knowledge. However, when she tried to describe the scene in the picture with the sentence “*zhe ge deng zi dao le*” (“This stool fell down”), she said, “*ze deng ze de dao le*.” Although the first part of her utterance “*ze deng ze de*” makes no sense in Chinese, the pronunciation sounds similar to the target “*zhe ge deng zi*,” in which she retained most final vowels, so people could easily guess her meaning. Another example of phonological errors is that the patient mispronounced “*huo hua ma*” (which does not make sense in Chinese) instead of “*huo ha ma*” (“live toad”) in a repetition task. When the patient described her condition and wanted to express the meaning that “the herbal medicine is burnt off,” she said, “the herbal medicine has gone bad,” which is a semantic error. All these errors were well-articulated without apraxia of speech.

She revisited the hospital for a follow-up consultation on 3 January 2020. Her relatives reported that the patient's speech was more fluent than before, and both word-finding difficulty and anxiety symptoms were improved. However, due to the impact of the coronavirus epidemic, the patient had not been actively seen since then.

The patient was readmitted to our department on 8 April 2021. According to her relatives, she deteriorated so markedly in word-finding that she usually could only say the first half of one sentence. In addition, her memory function declined as the disease progressed, so she frequently forgot what she was supposed to pick up when trying to get something. Sometimes the patient failed to find the things right in front of her, such as her cell phone. She also lost her way when going out. These symptoms suggest that her visual perception may be impaired, and so is her visuospatial ability. Even though the patient still had a clear knowledge of her condition. For example, she knew that she was forgetful and always stayed at the stove when cooking.

Her symptoms were obviously alleviated. However, the medication efficacy diminished after 1 year. Thorough assessments further evidenced her worsened word-finding and salient repetition difficulty. The same experienced rater performed and reported the neurolinguistic and neuropsychological evaluations. The patient scored 13 on the MMSE assessment and had a score on the NPI of 1, CDR of 0.5, and ADL of 29. The second ABC evaluation revealed a significant deterioration in repetition and calculation. Apart from that, the scores for other tasks were roughly the same (Table 2).

On 25 April 2021, another MRI examination was performed at the hospital (Figure 1). Two neuroimaging specialists rated the patient's cortical atrophy. Compared with the MRI in 2019, the second one revealed a further widened left lateral sulcus and asymmetric atrophy of the temporal lobes, with the most significant widening in the posterior part of the left insula. There was an increase in focal hyperintensity on T2 weighted images in the subcortical white matter of bilateral frontal-parietal lobes and paraventricular white matter. Significant HV atrophy was detected, with III-degree atrophy on the left hippocampal and II-degree atrophy on the right. All the above features suggest left posterior cerebral atrophy. Her physical examination was the same as before. The whole exome sequencing (WES) was also performed on the patient. A novel splicing variant (c.1615 G>A, p.539 G>S) was reported. The mutation c.1615G>A was absent in the public database of gnomAD East Asia. It was predicted to be deleterious by silico software, including SIFT and PolyPhen-2. According to the American College of Medical Genetics and Genomics (ACMG) guidelines, this is classified as “variants of uncertain significance (VUS)” (PM2+PP2). The apolipoprotein E (APOE) genotype was ε3ε3, and no causative gene for AD was detected.

Combined with the previously described and current diagnostic methods, the patient's presentation at the initial visit in 2019 met the diagnostic criteria for LVPPA. According to Gorno-Tempini's classification of PPA and its variants (9), establishing a clinical diagnosis involves a two-step process. Patients should first meet essential PPA criteria, which require a prominent, isolated language deficit during the initial phase of the disease. Based on Mesulam's guidelines (10), her most prominent clinical feature is difficulty with language, which is the primary cause of impaired daily living activities. Aphasia is the most remarkable deficit at symptom onset and for the initial phases of the disease. Therefore, all the inclusion criteria were answered positively. Meanwhile, her pattern of deficits could not be better accounted for by other non-degenerative nervous systems, nor could her cognitive disturbance be better accounted for by a psychiatric diagnosis. No significant initial episodic memory, visual memory, and visuoperceptual impairments or behavioral disturbance were observed. Hence, the exclusion criteria were answered negatively.

Therefore, the patient further meets the diagnostic criteria for lvPPA (9). For the clinical diagnosis of lvPPA, as the core features, both impaired single-word retrieval in spontaneous speech and naming and impaired repetition of sentences and phrases are present. Other features are also clearly present, such as speech (phonologic) errors in spontaneous speech and naming, spared single-word comprehension and object knowledge, and absence of frank agrammatism. Furthermore, the patient shows predominant left posterior perisylvian atrophy on MRI, meeting the imaging-supported diagnosis for lvPPA.

## Discussion

We describe and explore the case from the following three aspects.

First, from the perspective of neurolinguistics and neuropsychological analysis, there is only a 2-point difference between the patient's scores on two MMSE assessments, indicating that her overall cognitive function was stable. The second NPI evaluation increased with a score of 1 for the “agitation/aggression” item, and the patient became more stubborn and difficult to get along with than before. Moreover, the second ADL rating was five points higher than the previous one. She is slightly degraded in her ability to shop, make phone calls, manage her money, cook meals, and be alone at home. Therefore, with the disease's progression, her personality disorder and executive function impairment were increasingly prominent, only second to language dysfunction.

In previous studies, it has been found that lvPPA is primarily distinguished by a word-finding difficulty in spontaneous speech and confrontation naming, related to a “word-on-the-tip-of-the-tongue” phenomenon, and by problems in the repetition and comprehension of sentences due to her deficits of short-term memory (11). In the first test, the patient's speech disorder was characterized by an intermediate state between fluent and non-fluent spontaneous speech, which was ascribed to a prominent impairment of word retrieval. However, the patient would immediately consent when the rater said the target words for her, indicating the relatively preserved input lexicon and object knowledge. Phonemic paraphasias accounted for most of her speech errors. There is no frank agrammatism. Her repetition impairment was evidenced by difficulty in repeating sentences of more than five Chinese characters. The error rate of repetition increased with the length of the sentence. The aforementioned symptoms fit the description of lvPPA from earlier investigations. The outcome of the second test was the same as the first, with the exception of her dramatically diminished calculation ability. Further analysis of the error types revealed that the patient could correctly answer all the addition calculation questions, indicating no problem with her memory and perception of the numbers. However, the patient completed the subsequent subtraction and multiplication calculations in an additive manner, which resulted in errors (e.g., 6–2 = 8, 6^*^7 = 13). She was unable to do division calculations. Another important phenomenon related to the calculation errors observed in this patient was that she repeatedly performed the first action she was instructed, regardless of the following different instructions from the rater. Sandson and Albert (12, 13) originally proposed three various forms of perseveration: recurrent, stuck-in set, and continuous. Each of them is linked to a specific neuroanatomical network and each is influenced by a different neurochemical profile. Recurrent perseveration is an unintentional, immediate, or delayed repetition of a previous response after an intervening stimulus, which is linked to left temporoparietal lesions (14) and to dysfunction in cholinergic systems, and caused by postactivation of normally inhibited memory traces (15). Furthermore, it is linked to working memory functions mediated by frontal regions (16, 17). In this case, recurrent perseveration is correlated with temporoparietal function impairment, consistent with the left posterior perisylvian atrophy found on MRI.

Sentence repetition was further aggravated, as shown by the difficulty in her repetition of sentences of more than four Chinese characters. When the patient experienced word-finding difficulty during the evaluating process, she could make correct judgments with the hints given by the rater, which indicated that the underlying language-related dysfunction in lvPPA may result from the impaired access to the input lexicon or from the actual damage to the output lexicon.

In agreement with previous research, these results have confirmed the extensive damage to the language network in this lvPPA case. Neurolinguistic assessments have shown severe repetition impairments (repetition 27/100), along with the deficits of mild naming (word naming 26/40), and auditory comprehension (true/false questions 44/60), suggesting the possible presence of lesions in the left superior temporal gyrus and the left posterior inferior parietal lobe, as well as the bilateral fusiform gyrus (18).

Previous evidence suggests that 50–67% of the patients with lvPPA have probable Alzheimer's disease (19). In other words, AD might be the most common underlying pathology. Accordingly, lvPPA might be further divided into an expansive variant due to Alzheimer's disease (lvPPA due to AD, LDA) and a more localized variant not related to Alzheimer's pathology (lvPPA due to non-AD, LDNA). Previous research has demonstrated that cognitive impairments in LDA cases go even deeper into the language system (18). In addition, the effects of LDA extend beyond the language system, affecting syntactic processing and phoneme sequencing, thereby causing semantic representations to degrade further and even resulting in ideomotor apraxia. Pathologically, LDA involves predominant atrophy of the left temporal-parietal junction and a wider range of cerebral cortex than LDNA, including the inferior parietal lobe and the middle posterior temporal lobe in the left hemisphere.

As far as the current case is concerned, the patient should be classified as LDA because her positive PIB-PET result has indicated the presence of AD pathology. Her neurolinguistic and neuropsychological assessments have unveiled that the impairment of cortical function is not limited to her language function but extends to her short-term memory, calculation, visuospatial perception, and executive function. Furthermore, the recurrent perseveration exhibited by the patient in her execution of both verbal instructions and computational tasks suggests further impairment of the temporal-parietal lobe function. In line with the previous study (20), LvPPA may involve cortical regions beyond language areas, such as the parietal and frontal lobes.

According to epidemiological studies, patients with AD usually have an average annual decline of 4–5 points in the MMSE (21). However, the cognitive performance of this patient remained relatively stable. Her second MMSE score was two points lower than the first in 20 months, and the CDR assessments remained unchanged at 0.5 points. Two ADL evaluations differed by 6 points. It is clear from her slow rate of deterioration decline that the combination of rivastigmine transdermal patch and memantine hydrochloride tablet had been effective in maintaining her cognitive function. Schaeverbeke et al. investigated whether cholinergic alterations occur in PPA variants using N-[11 C]-Methylpiperidin-4-yl propionate (PMP)-PET, and found that only lvPPA cases, especially the cases show AD pathology, demonstrated decreases in Acetylcholinesterase (AChE) activity levels compared with controls. No differences were found in the navPPA or svPPA cases compared with controls (22). The cholinergic deficit in lvPPA in this study provides a potential rationale for the off-label use of AChE inhibitors in lvPPA due to AD pathology. This is also confirmed by the clinical efficacy of the current patient. Nevertheless, the patient still showed significant deterioration in repetition and calculation; thus, in-depth studies are needed to determine which specific mechanisms are involved.

Second, from the perspective of imaging analysis, different cortical atrophies and pathological changes are linked to different variants of PPA. With the imaging features of svPPA in the anterior and ventral temporal lobes and that of navPPA in the left inferior frontal and insula (23), it is crucial to identify abnormalities in the left temporoparietal junction area for the diagnosis of lvPPA. For the patient in this study, the analysis of MRI scans has shown the widened Sylvian fissures, suggesting the atrophy of the temporal lobe, which was more prominent on the left, as the initial detectable structural change in lvPPA. Furthermore, 20 months apart, the bilateral Sylvian fissure was further widened, with the most remarkable widening in the posterior part of the left insula. Significant hippocampal atrophy was also observed, suggesting that as the disease progresses, the temporal lobes and hippocampus could atrophy further bilaterally, and the atrophy is more pronounced on the left side.

Third, in addition to the above two aspects, WES identified the mutation of the MAPT gene. MAPT mutation is correlated with behavioral variant frontotemporal dementia, navPPA, svPPA, corticobasal degeneration, and progressive supernuclear palsy, while PGRN mutation may lead to lvPPA (24). According to our search of the relevant literature in the last decade, no case of MAPT mutation leading to lvPPA has been reported. This patient met the diagnostic criteria for clinical lvPPA and was found to have a progressive decline in short-term memory and visuospatial ability during follow-up, which does not support the diagnosis of frontotemporal dementia. The impact of the MAPT gene mutation on this patient is yet unknown. Further longitudinal and Tau-PET studies, even with pathological results, would be valuable.

## Conclusion

This article reports a typical Chinese-speaking patient with LDA due to AD. The follow-up of clinical manifestations and neurolinguistic characteristics confirms that, in addition to the linguistic features, lvPPA may be accompanied by the involvement of multiple cognitive domains with the progression of the disease, providing insight into the pathological mechanisms of this disease. Moreover, this patient had a mutation in the MAPT gene. Despite the fact that we are yet unsure of the exact role that this mutation plays in the patient's condition, this case provides us with proof that there may be an association between the MAPT mutation and LDA. The patient was nearly illiterate and unable to write or read; therefore, part of the linguistic profile cannot be analyzed. Since the evidence presented by one case is limited, further research is required to gather more thorough and convincing information.

## Data availability statement

The datasets presented in this article are not readily available because of ethical and privacy restrictions. Requests to access the datasets should be directed to the corresponding authors.

## Ethics statement

Written informed consent was obtained from the individual(s) and/or minor(s)' legal guardian/next of kin for the publication of any potentially identifiable images or data included in this article.

## Author contributions

BH: interpreting the data, drafting the manuscript, and revising the content. XW: drafting the manuscript and revising the content. BJ: collecting and processing the magnetic resonance imaging data. LK: collecting the neurolinguistic and neuropsychological assessment data. HH: collecting and processing the radioactive imaging data. JZ: collecting, analyzing, interpreting clinical data, and taking responsibility for conducting research and final approval. All authors contributed to the article and approved the submitted version.

## Conflict of interest

The authors declare that the research was conducted in the absence of any commercial or financial relationships that could be construed as a potential conflict of interest.

## Publisher's note

All claims expressed in this article are solely those of the authors and do not necessarily represent those of their affiliated organizations, or those of the publisher, the editors and the reviewers. Any product that may be evaluated in this article, or claim that may be made by its manufacturer, is not guaranteed or endorsed by the publisher.
